# SynSpine: an automated workflow for the generation of longitudinal spinal cord synthetic MRI data

**DOI:** 10.3389/fninf.2025.1649440

**Published:** 2025-12-17

**Authors:** Marco Ganzetti, Paola Valsasina, Frederik Barkhof, Maria A. Rocca, Massimo Filippi, Ferran Prados, Licinio Craveiro

**Affiliations:** 1 F. Hoffmann-La Roche Ltd., Basel, Switzerland; 2 Neuroimaging Research Unit, Division of Neuroscience, IRCCS San Raffaele Scientific Institute, Milan, Italy; 3 Queen Square Institute of Neurology, University College London, London, United Kingdom; 4 Department of Radiology and Nuclear Medicine, Amsterdam UMC, Vrije Universiteit, Amsterdam, Netherlands; 5 Vita-Salute San Raffaele University, Milan, Italy; 6 Department of Neurology, IRCCS San Raffaele Scientific Institute, Milan, Italy; 7 UCL Hawkes Institute, Department of Medical Physics and Biomedical Engineering, University College London, London, United Kingdom; 8 e-Health Center, Universitat Oberta de Catalunya, Barcelona, Spain

**Keywords:** synthetic data, artificial data, MRI, spinal cord, atrophy, simulation, digital phantom

## Abstract

**Background:**

Spinal cord atrophy is a key biomarker for tracking disease progression in neurological disorders, including multiple sclerosis, amyotrophic lateral sclerosis, and spinal cord injury. Recent MRI advancements have improved atrophy detection, particularly in the cervical region, facilitating longitudinal studies. However, validating atrophy quantification algorithms remains challenging due to limited ground truth data.

**Objective:**

This study introduces SynSpine, a workflow for generating synthetic spinal cord MRI data (i.e., digital phantoms) with controlled levels of artificial atrophy. These phantoms support the development and preliminary validation of spinal cord imaging pipelines designed to measure degeneration over time.

**Methods:**

The workflow consists of two phases: (1) generating synthetic MR images by isolating, extracting and scaling the spinal cord, simulating atrophy on the PAM50 template; (2) performing non-rigid registration to align the synthetic images with the subject’s native space, ensuring accurate anatomical correspondence. A proof-of-concept application utilizing the Active Surface and Reg methods implemented in Jim demonstrated its effectiveness in detecting atrophy across various levels of simulated atrophy and noise.

**Results:**

SynSpine successfully generates synthetic spinal cord images with varying atrophy levels. Non-rigid registration did not significantly affect atrophy measurements. Atrophy estimation errors, estimated using Active Surface and Reg methods, varied with both simulated atrophy magnitude and noise level, exhibiting region-dependent differences. Increased noise led to higher measurement errors.

**Conclusion:**

This work presents a novel and modular framework for simulating spinal cord atrophy data using digital phantoms, offering a controlled setting for testing spinal cord analysis pipelines. As the simulated atrophy may over-simplify *in vivo* conditions, future research will focus on enhancing the realism of the synthetic dataset by simulating additional pathologies, thus improving its application for evaluating spinal cord atrophy in clinical and research contexts.

## 1 Introduction

Spinal cord (SC) atrophy is an important biomarker for assessing disease progression in various neurological conditions, including multiple sclerosis (MS) (Rocca et al., 2005; Schlaeger et al., 2014; Kearney et al., 2015; Bischof et al., 2022; Tsagkas et al., 2022), amyotrophic lateral sclerosis (ALS) (Cohen-Adad et al., 2013; El Mendili et al., 2019; Wendebourg et al., 2024), and SC injury (SCI) (Lundell et al., 2011; Trolle et al., 2023; Seif et al., 2018, 2019, 2022).

In MS, SC atrophy is particularly prominent in progressive forms of the disease and is considered among the strongest predictor of clinical disability (Tsagkas et al., 2018; Mina et al., 2021). It progresses significantly over time at different rates, with higher rates associated with clinical worsening (Rocca et al., 2019). In addition, SC atrophy seems to be unrelated to cerebral atrophy suggesting partially independent patterns of neurodegeneration in these two compartments (Cohen et al., 2012; Ruggieri et al., 2015). In ALS, a notable reduction in the cross-sectional area (CSA) of the cervical SC has been reported in patients compared to healthy individuals (Barry et al., 2022). The extent of cervical atrophy has been found to progress over time, to be predictive of shorter life expectancy in ALS patients and found to correlate with functional deficits (de Albuquerque et al., 2017; Grolez et al., 2018). In SCI, there is a rapid and progressive degeneration of the SC, which is further aggravated by a neuro-immune response (Azzarito et al., 2020; Van Broeckhoven et al., 2021).

Recent advancements in MRI technology have greatly facilitated acquisition of detailed images of the SC, especially in the cervical region. Accurate measurement of annual atrophy rates as low as a few percent is now possible, even in short-term studies with moderate sample size, through semi-automated analysis on images obtained at C1, C2, or C2–C5 and beyond (Valsasina et al., 2015; Rocca et al., 2019).

In the last decade, several research groups have developed different methods for measuring atrophy, utilizing segmentation techniques based on the contrast between the SC tissue and the surrounding cerebrospinal fluid (CSF) in MR images. Among the most popular are a semi-automatic method based on an active surface (AS) model (Horsfield et al., 2010) and two fully-automated methods based on either propagation segmentation (PropSeg) (De Leener et al., 2014) or on convolutional neural networks (Deepseg) (Gros et al., 2019), respectively. Both PropSeg and Deepseg are implemented in the Spinal Cord Toolbox (SCT) (De Leener et al., 2017). The approach of extrapolating longitudinal changes from cross-sectional measurements involves numerically subtracting CSAs obtained separately at two different time points. However, the relatively high measurement noise and low reproducibility associated with specific segmentation-based methods when measuring the small structure of the SC can affect the accuracy and reliability of the results (Prados and Barkhof, 2018). To overcome the obstacles and enhance the assessment of SC atrophy, registration-based techniques have been developed, such as the Generalized Boundary Shift Integral (GBSI) (Prados et al., 2020), Reg (Valsasina et al., 2022) and recently SIENA-SC (Luchetti et al., 2024). Unlike segmentation-based techniques, GBSI involves capturing intensity changes in the cord profile over time. Reg consists in a refinement of the AS method including accurate, slice-wise registration between time points, while SIENA-SC is an adapted version of the original SIENA method (Smith et al., 2002) designed to directly calculate the percentage of SC volume change over time. These methods have shown to result in more reliable and consistent measurements of longitudinal changes compared to “purely” cross-sectional ones (Prados et al., 2020).

In the context of MS, there is compelling evidence suggesting the presence of subclinical disease progression characterized by SC atrophy (Mina et al., 2021), which occurs before the emergence of clinical worsening (Bischof et al., 2022). It is theorized that the loss of SC volume initiates during the early phase of the disease and precedes the clinical signs of progression (Zeydan et al., 2018; Rocca et al., 2019). For this reason, there is a recognized need to develop reliable imaging analysis techniques that can be automated for use in clinical trials. Such advancements would be valuable in assessing the effectiveness of treatments aimed at slowing down the neurodegenerative mechanisms of progression (Ontaneda et al., 2023).

The concept of simulated brain imaging methodologies is well established for verifying and validating novel methods and protocols (Stöcker et al., 2025). While earlier studies used physical phantoms (Amiri et al., 2019), digital phantoms that provide more accurate and more realistic models to simulate MRI studies are increasingly being utilized (Alfano et al., 2011). Recent studies that took advantage of digital phantoms, or simulated brain imaging methodologies, include the study by Prados et al. (2020), which involved an anisotropic axial shrinkage on the cord-straightened image, or the study by Bautin and Cohen-Adad, which used digital phantoms to rescale T1-weighted (T1-w) images isotropically using different scaling factors (Bautin and Cohen-Adad, 2021). So far, these approaches served as valuable resources for evaluating the performance of various algorithms. However, one notable limitation of these assessments is the use of global scaling, which resizes all anatomical structures proportionally. In a realistic scenario, SC volume decreases only in its soft tissues, but not the surrounding bones and muscles. In the case of segmentation-based methods, where each follow-up scan is treated as an independent readout, this scaling approach might be considered acceptable. However, for registration-based methods, which rely heavily on the registration between scans, the global scaling effect is typically canceled out in the processing pipeline.

The primary objectives of this study are twofold. Firstly, we aim to establish a comprehensive workflow for generating synthetic longitudinal SC MRI data overcoming the above-mentioned limitations, thus incorporating an artificial rate of cord atrophy but at the same time being suitable for testing both on segmentation-based and registration-based methods. Secondly, we seek to conduct a proof-of-concept evaluation of two well-established methods for the quantification of changes in the upper cervical cord area over time. By accomplishing these objectives, we aim to enhance our understanding of SC analysis techniques and contribute to the development of improved diagnostic tools. In accordance with these objectives, the present work focuses on demonstrating the feasibility and reproducibility of the proposed workflow rather than capturing population-level variability in SC anatomy. The simulated atrophy provides a controlled framework for testing and validating analysis pipelines.

## 2 Materials and methods

**FIGURE 1:**
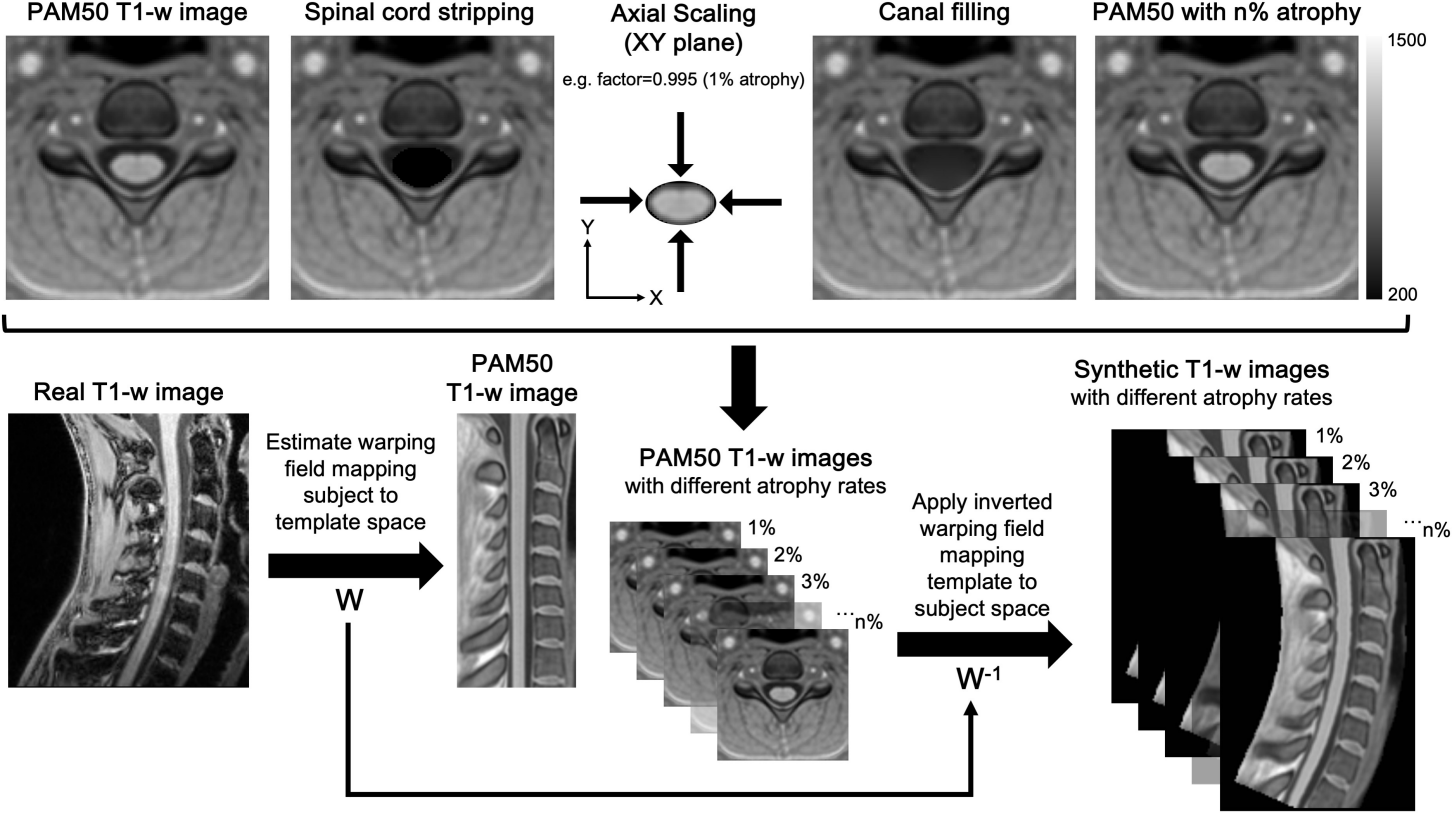
Workflow for the generation of synthetic MR images of the spinal cord. The workflow for generating and registering synthetic spinal cord images consists of two main phases: generation of synthetic MR images in template space (top) and the registration of the images from template space to subject native space (bottom). Phase 1: Firstly, the spinal cord was excised from the image (spinal cord stripping), and the excised region was filled using MATLAB’s *regionfill* function (canal filling), which interpolates pixel values based on the surrounding CSF. Secondly, the excised spinal cord was in-plane scaled (axial scaling) to simulate different atrophy levels and resliced using trilinear interpolation to minimize artifacts. Finally, the scaled spinal cord was superimposed onto the canal-filled images, creating synthetic templates with varying levels of simulated atrophy (e.g., PAM50 with n% atrophy). Phase 2: The non-rigid registration (W) was computed to ensure an accurate mapping of vertebral levels between the subject and the template, thus allowing a precise correspondence between the two images. Eventually, the inverted deformation (W-1) was applied to all synthetic images, transforming them into the subject’s native space and aligning their anatomical features with the subject’s spinal cord.

In this section, we outline SynSpine, a workflow for generating synthetic MR images of the spinal cord (i.e., digital phantoms) to simulate different levels of atrophy. Furthermore, we demonstrate the practical application of these digital phantoms by highlighting its potential in evaluating the performance of tools designed for analyzing SC atrophy. The generation of synthetic images was done using MATLAB R2023b (version: 23.2) and Spinal Cord Toolbox (SCT) v6.0 (De Leener et al., 2017). The SynSpine workflow is implemented within the MRTool software suite (version 1.5.0), which is freely available at https://www.nitrc.org/projects/mrtool/. To promote reproducibility and facilitate further research, all synthetic images used in this study are publicly available at https://www.nitrc.org/projects/synspine/. Figure 1 shows an overview of the processing pipeline.

### 2.1 Description of the workflow

#### 2.1.1 Generation of synthetic MR images in template space

The initial phase involves a sequence of image manipulation steps applied to the PAM50 SC template, which establishes a standardized reference image that is commonly utilized to facilitate population-based analyses. This multimodal MRI template of the SC and the brainstem is anatomically compatible with the ICBM152 brain template and uses the same coordinate system (De Leener et al., 2018). We used only T1-w images, as these are the most commonly used in clinical and research settings to evaluate tissue structures and determine important cord geometry measures, such as the CSA (Cohen-Adad et al., 2021b).

The procedure began with the dilation of the cord mask, a component of the PAM50 template, on each axial level. This was performed using the *imdilate* function in MATLAB, which takes advantage of a circular structuring element with a diameter of 4 pixels to achieve the dilation effect. This enlarged mask allowed for the cord to be excised from the image without compromising the cord boundaries. Once the cord was excised (SC stripping), the corresponding area was filled by employing MATLAB’s *regionfill* function. This function selects pixels of CSF that encircle the excised area and smoothly interpolates them inward, taking cues from the values at the region’s outer boundary. The function executes this by computing the discrete Laplacian over the specified area and solving the Dirichlet problem to seamlessly fill in the space (canal filling). Following the region-filling step, the extracted SC image underwent in-plane scaling to the desired sizes, and then it was resliced using trilinear interpolation to reduce artifacts at the interface between white matter and CSF. In the final step, the rescaled cord images were superimposed onto the image with the filled canal, creating multiple templates with simulated atrophy levels (PAM50 with n% atrophy). For a comprehensive understanding of the described processing steps, please refer to Figure 1 for a detailed depiction.

#### 2.1.2 Registration of synthetic images from template space to subject space

The second part of the workflow involves a series of image segmentation and registration steps utilizing SCT (De Leener et al., 2017) to align the PAM50 template (from which synthetic images with artificial atrophy rates are derived) to a real T1-w image. An initial segmentation delineates the SC in the subject’s image for precise template registration. SCT’s DeepSeg algorithm performs cord detection, centerline computation, image cropping, and final segmentation using a neural network applied to the cropped image (Gros et al., 2019). Next, vertebral labeling is performed (Ullmann et al., 2014) aligning vertebral levels between subject and template images. Two reference points, the first and last vertebral levels, are used to guide this alignment, ensuring accurate anatomical correspondence. Finally, multi-step non-rigid deformation adjusts the subject’s cord shape to match the template (De Leener et al., 2018). The first step handles large deformations; the second refines alignment. This transformation maps synthetic images from template space into the subject’s native space.

#### 2.1.3 Addition of noise

MR images can be degraded by thermal noise which negatively affects image processing task, such as registration and segmentation (Gudbjartsson and Patz, 1995). To investigate its impact on the workflow above, increasing levels of synthetic noise were added to the original data to simulate sequentially lower signal-to-noise ratio (SNR) conditions. While magnitude images in MRI are assumed to have Gaussian noise, the non-linear mapping from real and imaginary images creates a Rician noise distribution. It is also assumed that Gaussian noise in these images has equal standard deviation. For the purpose of this simulation, images have been produced with escalating degrees of noise (percentage relative to the median intensity of the SC) according to the following formula:


$$N\,I\,\sqrt{n^{2}+{\left(n+I\right)}^{2}}$$

**FIGURE 2:**
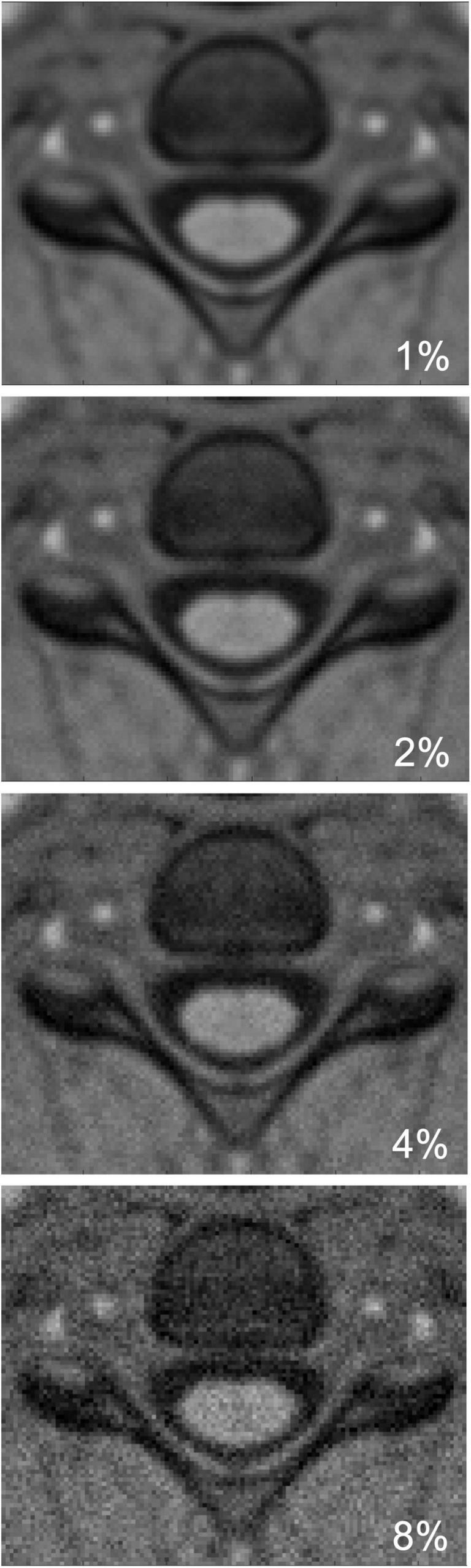
Simulated images with different levels of Rician noise on an axial slice from PAM50 template. From top to bottom: 1, 2, 4, and 8% added noise.

With NI and I as the image with added noise and the noise-free image, respectively. n is a tridimensional array of random numbers drawn from the normal distribution with mean equal to zero and standard deviation: σ_l = n_l⋅m_sc. n_l refers to the specific noise level and m_sc refers to the median SC intensity (extracted using the PAM50 cord mask). Figure 2 shows the synthetic PAM50 template image with 1, 2, 4, and 8% added Rician noise.

### 2.2 Method validation and statistics

**FIGURE 3:**
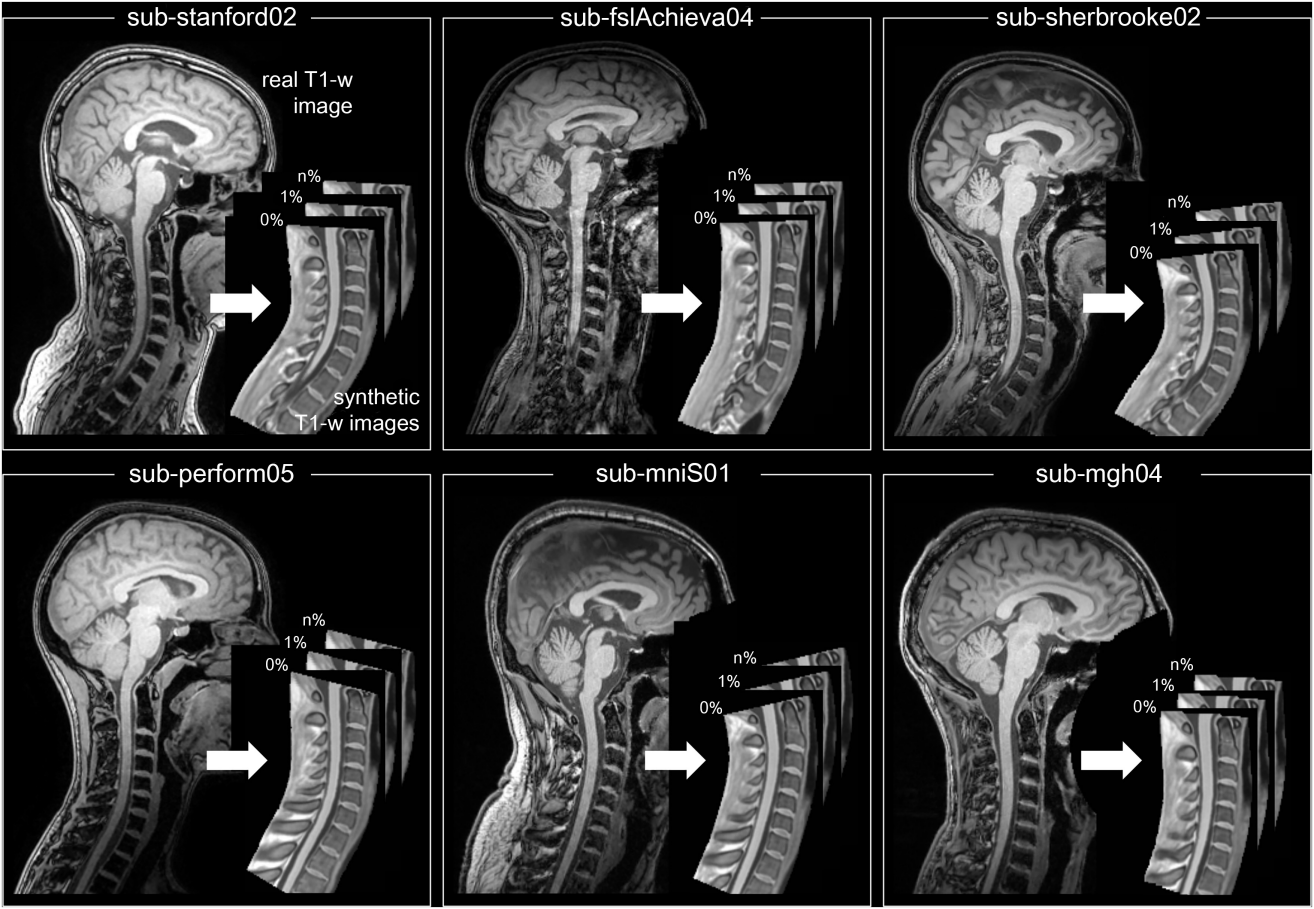
Example of the workflow output obtained for the six randomly selected subjects from the spine generic dataset. In each panel, the subject real MRI image is shown on the left and the respective synthetic images with different degree of simulated atrophy (in percentage) on the right.

In order to evaluate this novel workflow, a total of six subjects were randomly chosen from the open-access quantitative MRI dataset spine generic (Cohen-Adad et al., 2021b). This dataset consists of participants from various centers who underwent scanning following a predefined protocol (Cohen-Adad et al., 2021a). The data was collected, organized, and analyzed using a well-documented procedure, which can be found at https://spine-generic.rtfd.io. Out of all the sequences available in the dataset, the defaced 3D sagittal T1-w image was utilized for the purpose of validation. Table 1 provides detailed information regarding the demographics and scanner parameters for the selected subjects, and Figure 3 shows and example of the workflow output obtained for the six randomly selected.

**TABLE 1:** Baseline demographics.

Subject ID	Sex	Age	Height (cm)	Weight (kg)	Institution	Scanner	Receiver coil
sub-fslAchieva04	F	26	168	63	Santa Lucia Foundation IRCCS, Rome, Italy	Philips Achieva	NA
sub-mgh04	M	28	183	79	Athinoula A. Martinos Center for Biomedical Imaging, Charlestown, MA, USA	Siemens Skyra	64ch + spine
sub-mni01	M	37	174	76	McConnell Brain Imaging Centre, Montreal Neurological Institute, Canada	Siemens Prisma-fit	64ch + spine
sub-perform05	F	28	167	54	Concordia University, Perform Center, Montreal, Canada	GE MR750	NA
sub-sherbrooke02	F	32	167	51	Center d’imagerie moléculaire de Sherbrooke, Sherbrooke, Canada	Philips Ingenia	16ch head/neck + 12ch posterior
sub-stanford02	F	39	163	54	Richard M. Lucas Center, Stanford University School of Medicine, Stanford, CA, USA	GE MR750	16ch neurovascular

To evaluate the overall workflow, we conducted a visual assessment of the impact of artificial scaling on intensity distributions within the SC mask provided with the PAM50 template. This was achieved by plotting the pairwise distributions between the intensity of the atrophy-free image and each image exhibiting incremental levels of atrophy. To evaluate the accuracy of reinserting rescaled cord images onto the canal-filled PAM50 template, as well as the effectiveness of trilinear interpolation in minimizing artifacts at the CSF/SC interface, we performed a quantitative analysis of the partial volume effect at the cord boundary. For each simulated atrophy level, a boundary ring mask was generated by computing the difference between dilated and eroded versions of the corresponding stripped SC mask. Dilation and erosion were performed using MATLAB’s *imdilate* and *imerode* functions, respectively, with a circular structuring element of 1-pixel diameter. This process produced a 2-pixel–wide ring in the axial plane, capturing the CSF/SC transition zone specific to each rescaled cord image. The ring mask for each simulated atrophy level was then applied to extract intensity values from the corresponding canal-filled PAM50 template. These values were used to quantify changes at the boundary region across simulated atrophy levels. Independent-sample *t*-tests were conducted to compare the original (non-atrophied) PAM50 template with each rescaled version, providing a statistical assessment of potential differences in boundary intensity resulting from the reinsertion process. To quantify the magnitude of these differences, Cohen’s *d* was calculated as an estimate of effect size.

Subsequently, to quantify the influence of non-rigid registration on the simulated atrophy as defined by the PAM50 template, we computed the voxel-wise differences between the intensity distributions extracted from the PAM50 SC mask on the atrophy-free image and the corresponding images with varying degrees of atrophy. This analysis was performed for both the PAM50 template and the simulated images of each subject. A two-sample Kolmogorov-Smirnov (KS) test was performed to compare the cumulative differential distributions derived from the PAM50 template with those from the subject-specific images featuring incremental levels of simulate atrophy. The KS is a nonparametric test that compares the cumulative distributions of two independent samples (PAM50 vs. synthetic subject). The test statistics D represents the maximum difference between the two cumulative distributions and it ranges between 0 and 1, where 0 occurs if the two distributions are identical, and 1 if the two are completely distinct. To assess the impact of the anatomy, the analysis was performed for each subject at three cervical levels: (i) cord segment between upper C1 extremity and C5/C6 intervertebral disk (“C1C5”); (ii) cord segment between upper C1 extremity till C2/C3 intervertebral disk (“C1C2”); and (iii) cord segment between C2/C3 and C5/C6 intervertebral disks (“C2C5”). In order to have accurate definitions of these vertebral landmarks, spinal level anchoring was defined at the mid-disc level between vertebrae using SCT labeling tools.

### 2.3 Proof of concept

The potential of using synthetic images to assess the effectiveness of methods for measuring SC atrophy was investigated. Specifically, the Active Surface (AS) and Reg methods within the Jim software (Xinapse Systems, Colchester, United Kingdom)^1^ were chosen for this assessment. The AS algorithm applies a semiautomatic active surface model, which is based on SC surface parametrization, yielding reproducible measurements of cord cross-sectional areas (Horsfield et al., 2010). In contrast, the Reg method extends the AS approach by incorporating accurate slice-wise registration between time points, enabling improved assessment of SC atrophy in longitudinal studies (Valsasina et al., 2022). The performance of both methods was then tested for increasing levels of cord atrophy (0.5, 1, 1.5, 2, 3, 4, 5, 6 and 10%) and noise (i.e., 1, 2, 4, 8%, and no noise). CSA was calculated on synthetic images in two different cervical segments (C1C2 and C2C5, as defined above). Cord atrophy is calculated by comparing the numerical differences in CSA between two time points: one with a specified simulated atrophy rate and the other serving as a reference with no atrophy applied. Notably, atrophy and noise were simulated across the entire SC in a single procedure, rather than dividing the process into two separate parts for C1C2 and C2C5. The performance of both methods was evaluated using both simulated images based on the PAM50 template and synthetic images derived from six subjects extracted from the spine generic dataset. For the PAM50 analysis, the error was determined as the difference between the measured and simulated atrophy values for each combination of noise and atrophy. In the case of the spine generic dataset, the error was assessed using the Root Mean Squared Error (RMSE) across the six subjects. This approach quantifies the overall deviation, with lower RMSE values indicating higher accuracy. In addition, the association between AS and Reg was evaluated using Pearson’s correlation coefficient (*r*) for each subject, separately for the cervical segments C1C2 and C2C5, and across all noise levels.

## 3 Results

### 3.1 Assessing effectiveness of the workflow to generate synthetic SC atrophy images

**FIGURE 4:**
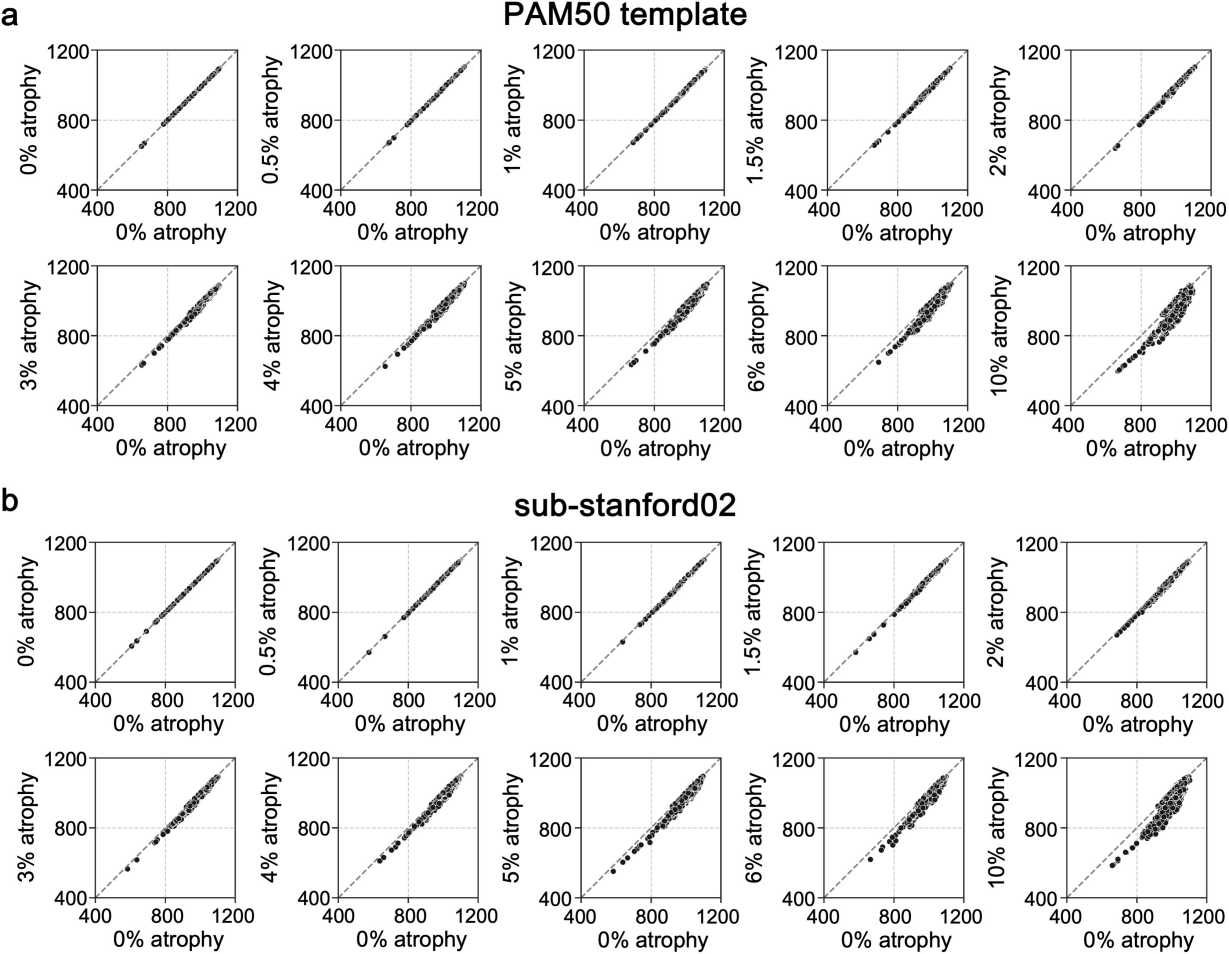
Characterization of the intensity distribution for different levels of simulated atrophy in comparison to 0% atrophy for PAM50 **(a)** and a representative subject from the spine generic dataset **(b)**. The intensity distributions refer to the cervical segment from C1 to C5.

To evaluate whether SynSpine was able to generate images with simulated levels of atrophy, the distribution of intensities of the original SC binary mask (without atrophy, 0%) was compared to the corresponding intensities of the SC with different levels of induced artificial atrophy (0.5, 1, 1.5, 2, 3, 4, 5, 6 and 10%) in the PAM50 template (Figure 4a), and each of the 6 subjects (results for a representative subject [sub-stanford02] are shown in Figure 4b; for all remaining subjects, please refer to Supplementary Figure 1). In both cases, the varying degrees of induced atrophy can be observed, as demonstrated by the deviation from the diagonal line. This deviation is proportional to the degree of induced atrophy.

### 3.2 Validation of SC rescaling and reinsertion: preservation of boundary integrity and intensity profiles

**FIGURE 5:**
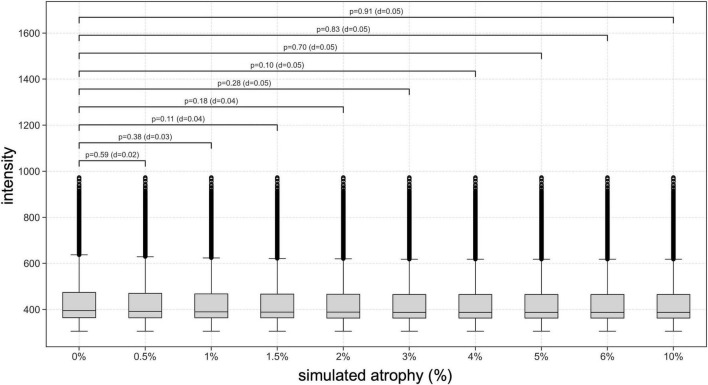
Validation of cord rescaling and reinsertion in the PAM50 template: preservation of CSF/SC boundary integrity and intensity profiles. Intensity values were extracted within a 2-pixel–wide boundary ring mask applied to the canal-filled PAM50 templates. This mask effectively captured the CSF/SC transition zone, providing a sensitive region for partial volume assessment. Boxplots show the distribution of boundary intensity values across increasing levels of simulated atrophy (0–10%). Independent-sample *t*-tests revealed no significant differences (*p* > 0.05) in boundary intensity between the original (non-atrophied) and rescaled templates at any atrophy level. Corresponding Cohen’s *d* values indicated negligible effect sizes (*d* ≤ 0.05), confirming the absence of meaningful deviations in intensity profiles. These findings demonstrate that the rescaling and reinsertion process preserves both the anatomical and intensity continuity of the CSF/SC interface, validating the robustness of the simulation approach.

To evaluate the impact of cord rescaling and reinsertion on the CSF/SC boundary, intensity values were extracted within the 2-pixel–wide boundary ring mask applied to the canal-filled PAM50 templates. Visual inspection of axial slices confirmed that the mask effectively captured the transition zone, providing a sensitive region for partial volume analysis. Quantitative analysis of these intensity values revealed no significant deviations between the original (non-atrophied) template and the rescaled versions across all simulated atrophy levels. Independent-sample *t*-tests showed no significant differences in boundary intensity between the original template and any rescaled version (*p* > 0.05), indicating that reinsertion did not introduce detectable artifacts or distortions at the CSF/SC interface (Figure 5). Trilinear interpolation during reinsertion maintained smooth intensity transitions, minimizing partial volume effects and preserving anatomical realism at the cord boundaries. Assessment of Cohen’s d further confirmed the absence of meaningful differences in intensity profiles. These results validate that the simulation and reinsertion process preserves both the structural integrity and intensity characteristics of the SC in the PAM50 template across varying levels of simulated atrophy.

### 3.3 Impact of non-rigid registration

Additionally, to evaluate the impact of the non-rigid registration used to transform the artificial image from PAM50 template space to the original subject space (as described in Figure 1), the intensity differences between the SC without atrophy and the cord with a specific atrophy rate were computed for both the PAM50 template and the non-linearly registered PAM50 template in subject space. Table 2 shows the two-sample KS test statistic (D) for different simulated atrophy values computed for each subject at three cervical levels (C1C5, C1C2, and C2C5). The D value reported in the table represents the maximum difference between the two cumulative distributions. No significant differences (*p*> 0.05) were observed at any cervical level for any subject (*p*-values are reported in Supplementary Table 1).

**TABLE 2:** Impact of the non-rigid registration from template to subject space on simulated atrophy.

Cervical	Subject	Two-sample Kolmogorov-Smirnov test statistics (D) by percent simulated atrophy								
level	ID	0.5%	1%	1.5%	2%	3%	4%	5%	6%	10%
C1C5	sub-fslAchieva04	0.013	0.014	0.012	0.012	0.014	0.014	0.014	0.013	0.016
	sub-mgh04	0.014	0.015	0.019	0.021	0.023	0.021	0.022	0.022	0.021
	sub-mni01	0.02	0.019	0.021	0.02	0.021	0.022	0.021	0.021	0.021
	sub-perform05	0.014	0.021	0.022	0.024	0.024	0.023	0.025	0.024	0.024
	sub-sherbrooke02	0.007	0.013	0.012	0.013	0.013	0.013	0.013	0.012	0.012
	sub-stanford02	0.007	0.019	0.02	0.021	0.023	0.021	0.022	0.023	0.022
C1C2	sub-fslAchieva04	0.033	0.042	0.038	0.042	0.044	0.047	0.046	0.048	0.048
	sub-mgh04	0.014	0.021	0.018	0.021	0.021	0.023	0.021	0.022	0.021
	sub-mni01	0.029	0.04	0.035	0.039	0.04	0.038	0.041	0.04	0.038
	sub-perform05	0.02	0.027	0.025	0.024	0.025	0.024	0.023	0.025	0.021
	sub-sherbrooke02	0.014	0.022	0.022	0.025	0.022	0.025	0.025	0.024	0.025
	sub-stanford02	0.025	0.024	0.03	0.028	0.029	0.027	0.028	0.029	0.027
C2C5	sub-fslAchieva04	0.012	0.019	0.022	0.023	0.019	0.021	0.021	0.021	0.021
	sub-mgh04	0.011	0.017	0.018	0.021	0.019	0.02	0.02	0.02	0.021
	sub-mni01	0.01	0.011	0.015	0.016	0.015	0.015	0.015	0.015	0.017
	sub-perform05	0.013	0.014	0.014	0.014	0.016	0.016	0.017	0.017	0.018
	sub-sherbrooke02	0.008	0.011	0.014	0.013	0.013	0.013	0.013	0.013	0.015
	sub-stanford02	0.008	0.015	0.018	0.016	0.017	0.017	0.017	0.019	0.017

Two-sample Kolmogorov-Smirnov test statistics (D) for different simulated atrophy values are reported for each subject at three cervical levels (C1C5, C1C2, and C2C5). This test is a nonparametric test that compares the cumulative distributions of two independent samples (PAM50 vs. synthetic subject). The D value reported in the table represents the maximum difference between the two cumulative distributions. The D test statistic is a distance metric between different empiric distributions and it ranges between 0 and 1, where 0 occurs if the two distributions are identical, and 1 if the two are completely distinct. No significant difference between the intensity distribution of the template and each subject distribution.

### 3.4 Proof of concept

**FIGURE 6:**
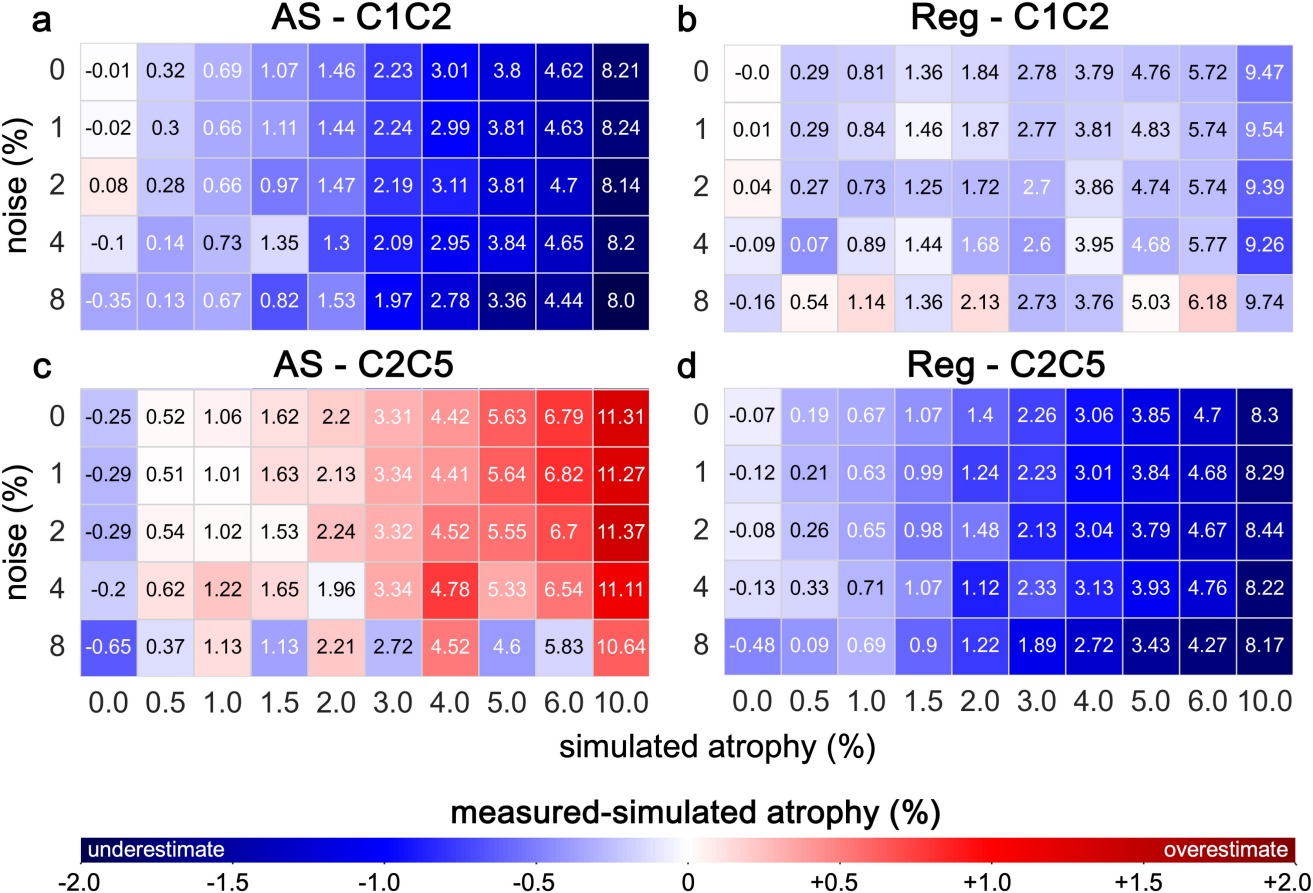
Estimated atrophy using Jim’s AS and Reg on synthetic images in the PAM50 template space. The numerical values in the color matrix denote the measured atrophy levels and the color shades visually represent the magnitude of the error (measured- simulated atrophy). The measured atrophy from CSA estimates is presented for various levels of simulated atrophy under different noise conditions. Analyses were conducted for the cervical segments C1C2 **(a,b)** and C2C5 **(c,d)**. A positive error denotes overestimation, while a negative error signifies underestimation of the expected atrophy level.

Overall, the estimated atrophy measurements using the Jim’s AS and Reg showed variable levels of agreement with the simulated ground truth values, depending on the degree of simulated atrophy, the level of noise and the anatomical level. In images based on the PAM50 template without added noise (Figure 6 and Supplementary Tables 2, 3), the measurement error for the AS method ranged from −0.01 to −1.79% at C1C2 level and from −0.25 to 1.31% at the C2C5 levels. For Reg method, the error ranged from 0 to −0.53% at C1C2 and from −0.05 to −1.7% at C2C5. Measurement error increased with higher levels of simulated atrophy across both cervical regions for both methods. For the AS method, atrophy was generally underestimated at C1C2 and overestimated at C2C5 relative to the ground truth. In contrast, the Reg method showed underestimation at both cervical levels, with a larger magnitude at C2C5 than at C1C2. At C1C2, Reg demonstrated superior accuracy compared with AS, exhibiting a smaller underestimation even as noise levels increased; however, this advantage was not observed at C2C5. Overall and expectedly, increasing levels of noise resulted in greater measurement error, but with no substantial differences. Interestingly, for the AS method at C2C5, measurement error decreased with increasing noise at higher simulated atrophy levels (≥5%), whereas for the Reg method, error increased under the same conditions.

**FIGURE 7:**
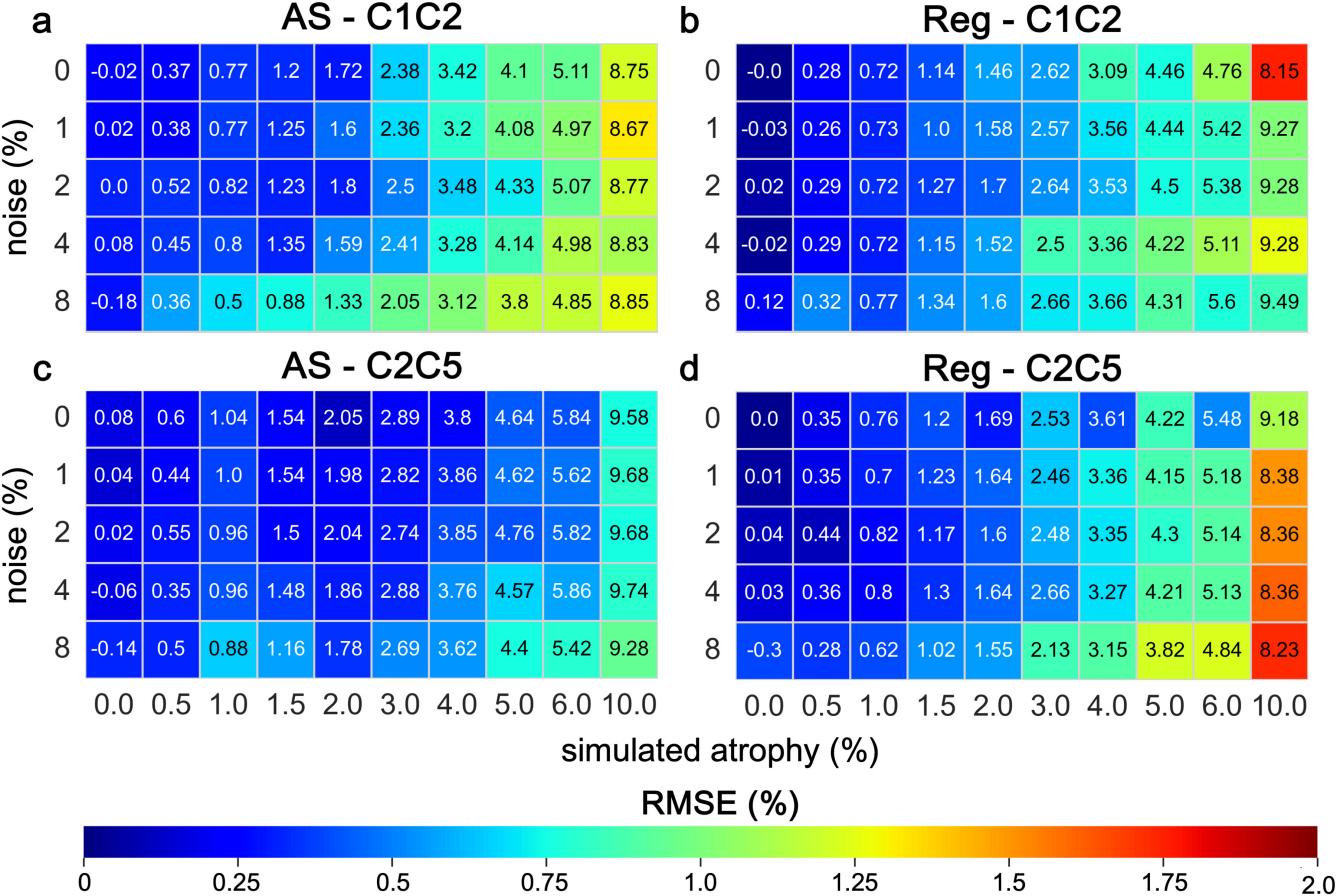
Averaged estimated atrophy using Jim’s AS and Reg on synthetic images generated from real T1-w images of six randomly selected subjects from the spine generic dataset. The numerical values in the color matrix denote the mean value of measured atrophy and the color shades visually represent the RMSE. The measured atrophy from CSA estimates is presented for various levels of simulated atrophy under different noise conditions. Analyses were conducted for the cervical segments C1C2 **(a,b)** and C2C5 **(c,d)**. RMSE quantifies the overall deviation, with lower values indicating higher accuracy.

**FIGURE 8:**
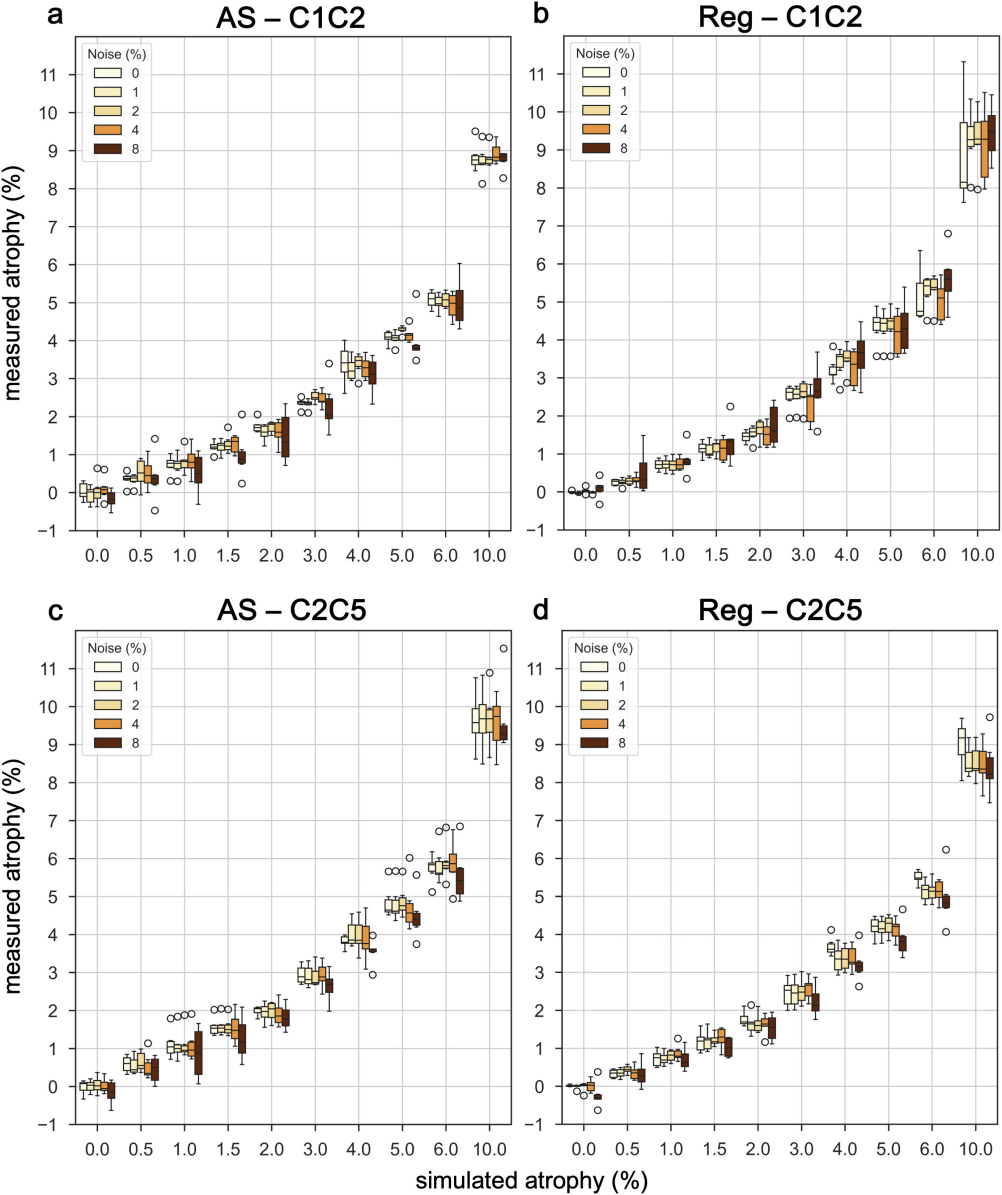
Performance of Jim’s AS and Reg on synthetic images generated from real T1-w images of six subjects. Measured atrophy from CSA estimates is reported for different values of simulated atrophy at different noise levels. The analysis was performed for the cervical segment C1C2 **(a,b)** and C2C5 **(c,d)**.

**FIGURE 9:**
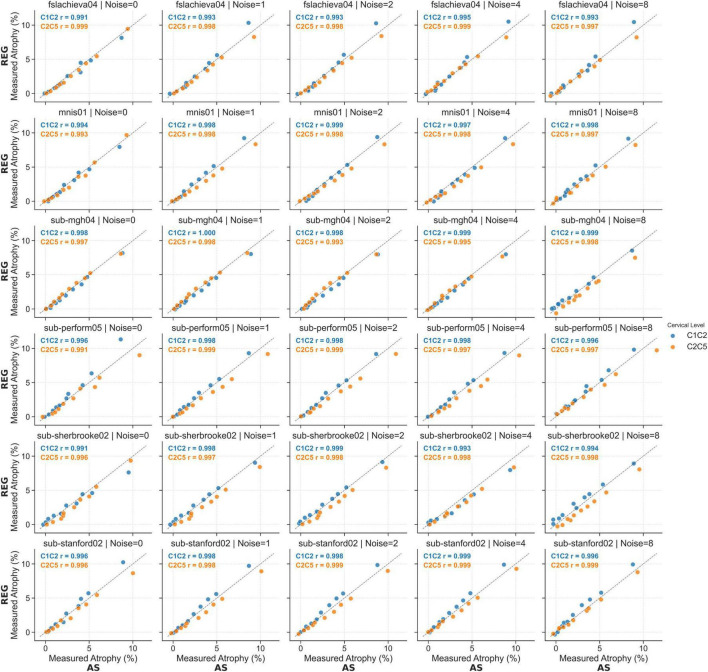
Comparison between Jim’s AS and Reg on synthetic images generated from real T1-w images. Measured atrophy estimated by the Reg method is plotted against atrophy measured by the AS method for synthetic images generated from six subjects. Each column corresponds to a different level of added Rician noise (0, 1, 2, 4, and 8%), and each row corresponds to a different subject. Each point represents one simulated atrophy level ranging from 0 to 10%. Pearson’s correlation coefficients (*r*) are reported for both cervical cord segments C1C2 (blue) and C2C5 (orange), demonstrating strong agreement between the two methods across all subjects and noise levels.

Results for the six subjects selected from the spine generic dataset were overall consistent with those observed for the PAM50 template (Figure 7 and Supplementary Tables 4, 5). The mean atrophy across the six subjects was computed along with the RMSE. In images without added noise, the RMSE for the AS method ranged from 0.21 to 1.22% at C1C2 and from 0.11 to 0.75% at the C2C5. For the Reg method, the RMSE ranged from 0.02 to 1.76% at C1C2 and from 0.03 to 1.12% at the C2C5. The measurement error increased as the simulated atrophy increased for both cervical segments. For both methods, greater inter-subject variability (i.e., larger quartile bounds) was observed with increasing levels of noise for both cervical levels (Figure 8). Additionally, at lower atrophy levels (<2%), the Reg method exhibited smaller inter-subject variability compared with the AS method. Overall, the level of agreement between the two methods across all noise levels and cervical SC segments was exceptionally high (Pearson’s *r* > 0.99) (Figure 9). The consistency of the correlation coefficients indicates that the two method remains highly robust, even when increasing levels of synthetic noise were introduced into the data. Across all subjects, both the C1C2 and C2C5 segments demonstrated near-perfect linear relationships between the measured and reference atrophy values, with minimal deviations from the identity line. Notably, the correlations for both cervical segments remained virtually unchanged across noise conditions, suggesting that the algorithm’s agreement is stable and resistant to noise-related degradation. Minor variability observed at the highest atrophy level (10%) did not substantially affect the overall accuracy or agreement between methods.

## 4 Discussion

In this study, we introduced SynSpine, a novel workflow for generating spinal cord digital phantoms which provide standardized ground truth data on spinal cord atrophy. The access to such ground truth data can help researchers in the initial steps of development and validation of analysis algorithms, providing a solid foundation for benchmarking.

### 4.1 Novelty of the workflow

This workflow addresses critical limitations of existing approaches like global scaling (Bautin and Cohen-Adad, 2021). While such scaling proportionally resizes the entire MRI image to simulate atrophy, our method exclusively applies rescaling to the SC itself. This targeted approach better replicates the real-world scenario, where atrophy is localized to the SC and does not affect surrounding structures, such as the spinal canal or vertebrae (Petrova et al., 2018). Methods relying on global scaling do not generate localized differences between consecutive scans, which are critical for algorithms that target region-specific atrophy patterns mainly driven by the image gradient at the CSF/SC interface (Prados et al., 2020). Additionally, while global scaling may still find utility in benchmarking cross-sectional analysis methods (Bautin and Cohen-Adad, 2021), its applicability is limited when localized intensity variations are a focus. The proposed method thus offers a broader and more flexible workflow that accommodates both cross-sectional and longitudinal studies.

### 4.2 Effectiveness of synthetic atrophy generation

The ability of the workflow to generate progressive and measurable atrophy was validated through intensity distribution comparisons (Figure 4). The observed deviations proportional to induced atrophy levels confirm the workflow’s capacity to simulate realistic morphological changes. Furthermore, the consistency of these results across subjects underscores the generalizability of the approach (Supplementary Figure 1). The absence of significant intensity differences at the CSF/SC boundary across all simulated atrophy levels demonstrates that the simulation preserves anatomical and intensity fidelity (Figure 5). The finding suggests that the implemented approach effectively minimizes interpolation-related artifacts and maintains realistic boundary transitions, which are critical for accurate partial volume representation. By ensuring smooth intensity gradients and structural continuity, the results provide confidence that simulated atrophy reflect genuine geometric changes rather than artificial intensity distortions. This supports the robustness of the framework for validating quantitative SC analysis methods under controlled conditions. In addition, non-rigid registration, a critical step in adapting synthetic images from the PAM50 template to individual subject spaces, showed no significant impact on the simulated atrophy rates. The influence of the registration was assessed using the two-sample KS test by evaluating differences in the cumulative intensity distributions between the PAM50 template in its native space and the registered template in individual subject spaces across varying simulated atrophy values (Table 2 and Supplementary Table 1). No significant differences were reported at any cervical level indicating that the transformation process preserves the integrity of the simulated atrophy, ensuring the reliability of downstream analyses. This finding is crucial for clinical translation, as the non-linear transformation enables subject-specific assessments without compromising the fidelity of the simulated data.

### 4.3 Proof of concept: Jim’s AS and Reg

The current solution effectively addresses the simulation of atrophy, providing a solid foundation for algorithm benchmarking. This is supported by our results of the proof-of-concept application of Jim’s AS and Reg methods to the generated synthetic dataset, which revealed an alignment between the measured atrophy values and the expected ones. Nonetheless, the analysis revealed certain trends in the method’s performance, influenced by factors such as noise levels, anatomical location, and varying degrees of atrophy.

When evaluating the AS method on the PAM50 template, measurement errors at the C1C2 and C2C5 levels showed an absolute increase as the simulated atrophy rates increased (Figure 6). However, the error exhibited contrasting trends: underestimation of the reference values with increasing atrophy at C1C2 but overestimation at C2C5 (Figure 6 and Supplementary Table 2). This discrepancy may reflect differences in anatomical complexity or sensitivity to induced atrophy at these levels. Interestingly, at C2C5, for higher atrophy rates (≥5%), the paradoxical reduction in measurement error with increasing noise levels may point to non-linear interactions between noise and atrophy effects. This phenomenon warrants further investigation to understand its underlying mechanisms, which could inform the development of noise-robust measurement methodologies. The overall underestimation of true atrophy and the greater measurement error observed at C1C2 compared to C2C5 suggests that this region may be more prone to measurement errors due to its smaller size and higher sensitivity to segmentation inaccuracies. In comparison, the Reg method demonstrated consistent underestimation of atrophy at both C1C2 and C2C5 levels, with a larger magnitude at C2C5 than at C1C2 (Figure 6 and Supplementary Table 3). The underestimation of atrophy using the Reg vs. AS method is consistent with previous findings (Valsasina et al., 2022). Notably, at C1C2, Reg exhibited superior accuracy relative to AS, producing smaller underestimation across all noise levels. This advantage, however, was not seen at C2C5, where Reg continued to underestimate atrophy while AS tended to overestimate at higher simulated atrophy rates. Moreover, Reg’s measurement error increased with higher noise levels, contrasting with the paradoxical decrease observed for AS at C2C5 under high atrophy conditions. These findings emphasize the need for refined measurement techniques or correction algorithms tailored to specific SC regions (Lukas et al., 2021).

The results from the synthetic images from the six subjects in the spine generic dataset generally aligned with those of the PAM50 template, demonstrating the workflow’s adaptability (Figure 7). Specifically, for the AS method, the tendency to underestimate CSA values at the C1C2 level underscores the significant impact of anatomical factors (Supplementary Figure 2), a bias that was not observed with the Reg method (Supplementary Figure 3). Overall, the general pattern observed across subjects indicates greater errors at higher atrophy rates, with errors further increasing as noise levels rise (Figure 7 and Supplementary Figures 2, 3). The results also revealed a pronounced increase in inter-subject variability, indicated by the expanding quartile bounds, as noise levels rise, regardless the cervical level (Figure 8). This suggests heightened sensitivity of measurements to noise, possibly explained by an increase in partial volume effect at the interface CSF/SC (Bautin and Cohen-Adad, 2021). This trend underscores potential biases introduced by noise and suggests the need for refined methodologies to ensure robust quantification in situation of low SNR. Such variability must be carefully accounted for in clinical applications, particularly in longitudinal studies where small changes in cord morphology are of diagnostic significance. Although inter-subject variability increased with added noise for both methods, Reg showed smaller variability at lower atrophy levels ( < 2%), which correspond to atrophy rates detected in most real-world studies. Despite these differences, agreement between AS and Reg remained exceptionally high across all noise conditions and cervical levels, reflecting robust, near-perfect linear relationships between methods (Figure 9). Even at the highest atrophy level (10%), minor deviations did not substantially affect overall accuracy or concordance between methods, indicating that both algorithms provide reliable and noise-resistant measurements across the upper cervical SC.

### 4.4 Limitations and future endeavors

Our methodology has several limitations that should be taken into account. Firstly, the synthetic images generated in our study do not fully mimic the biological variability encountered in real-life datasets and, therefore, it likely oversimplifies *in vivo* conditions. One such example is the fact that the digital phantoms generated by SynSpine do not currently incorporate any pathological features, such as SC lesions (commonly observed in diseases like MS) which are known to significantly influence the assessment of atrophy by confounding lesion intensities with those of normal structures (Gros et al., 2019). On the other hand, it was previously demonstrated that AS and Reg methods are not particularly sensitive to T1-hypointense SC lesions (Valsasina et al., 2018), suggesting a limited impact of this issue on the presented results. In addition, the current version does not more intricate pathological volume loss patterns, including asymmetric atrophy (Lundell et al., 2017). From an artifact perspective, another critical consideration is the absence of intensity inhomogeneities, which commonly appear as smooth intensity variations across MR images (Ganzetti et al., 2016), often along the superior-to-inferior axis (Gros et al., 2019). Such artifacts can pose challenges for the assessment of atrophy, particularly for intensity-based tools that rely on consistent intensity differences between baseline and follow-up scans for accurate quantification. Another common artifact in MRI imaging not currently modeled is patient movement, which can introduce significant challenges by causing blurring and aliasing of the MR signal, ultimately compromising image quality (Zaitsev et al., 2015). It should also be noted that the current work used only T1-w images. While this sequence is the most widely utilized in clinical and research settings for assessing tissue structures and determining critical cord geometry measures, such as the CSA, it is important to simulate atrophy using images acquired with different sequences—SynSpine can be easily adapted for other MRI contrasts. Finally, the SynSpine framework was developed using a single template (PAM50) and a small number of subjects. While this is not unusual when developing digital phantoms, further work should include generation of phantoms using different templates or atlas of more diverse cohorts that cover for example age and sex-specific atrophy gradients and thus increase the anatomic variability of the phantom and the generalizability of the simulations.

## 5 Conclusion

We have developed a framework that generates digital phantoms for spinal cord atrophy using MRI data acquired *in vivo*. The proposed method overcomes the inherent limitations of current methods which apply proportional scaling to an image (global scaling), by introducing instead a more anatomically realistic approach for synthetic data generation. This innovation enhances the ability to study and detect SC atrophy, particularly in longitudinal scenarios, while retaining the flexibility to support cross-sectional analyses. This framework is a first step to improve synthetic atrophy models as a tool to help develop and validate segmentation and registration-based cord analysis pipelines. Future work will focus on two main directions. First, we aim to enhance and expand the dataset to improve its realism and representativeness by incorporating greater anatomical variability, a wider range of acquisition protocols, and simulated pathological features such as focal lesions, SC compression, and various imaging artifacts. These improvements will increase the generalizability and clinical relevance of the results. Second, we will perform a comprehensive comparison of existing SC algorithms (e.g., GBSI, SIENA-SC) to systematically evaluate their accuracy, robustness to noise and atrophy, and sensitivity to regional differences. Together, these efforts will help identify the most reliable and context-appropriate methods for quantifying SC atrophy across diverse imaging conditions and populations.

To ensure transparency and facilitate further research, the complete source code is publicly available at https://www.nitrc.org/projects/mrtool/, enabling researchers to generate synthetic datasets from their own subject cohorts and to extend the approach to broader demographic, pathological, and morphological contexts.

## Data Availability

The synthetic images generated using the presented workflow are freely available at https://www.nitrc.org/projects/synspine/ under the Apache License 2.0 for unrestricted use, modification, and distribution.

## Footnotes

1
www.xinapse.com
