# Supplementary material for: SynSpine: an automated workflow for the generation of longitudinal spinal cord synthetic MRI data
Source: Front Neuroinform. 2025 Dec 17;19:1649440. doi: 10.3389/fninf.2025.1649440 (PMC12753887; doi:10.3389/fninf.2025.1649440)
Supplement: Supplementary file 1 [file Data_Sheet_1.docx]

Supplementary Material

**Supplementary Table 1.** Impact of non-rigid registration from template to subject space on simulated atrophy. Two-tailed Kolmogorov–Smirnov (KS) test p-values are reported for each subject at three cervical levels (C1C5, C1C2, and C2C5) across different simulated atrophy levels. No significant differences were observed between the intensity distributions of the template and the synthetic subject images (p > 0.05), indicating that the non-rigid registration preserves the underlying intensity characteristics while simulating atrophy.

For reference, the sample sizes correspond to the number of voxels within the respective masks for each subject and segment:

- **C1C5:** fslAchieva04: 4960, mgh04: 6214, mni01: 4989, perform05: 4281, sherbrooke02: 4392, stanford02: 4368
- **C1C2:** fslAchieva04: 1365, mgh04: 2146, mni01: 1507, perform05: 1259, sherbrooke02: 1291, stanford02: 1292
- **C2C5:** fslAchieva04: 4561, mgh04: 5323, mni01: 4545, perform05: 3924, sherbrooke02: 4027, stanford02: 4008

| **Cervical level** | **Subject ID** | **Two-tailed Kolmogorov–Smirnov (KS) test p-values by percent simulated atrophy** | | | | | | | | |
| --- | --- | --- | --- | --- | --- | --- | --- | --- | --- | --- |
|  |  | **0.5%** | **1%** | **1.5%** | **2%** | **3%** | **4%** | **5%** | **6%** | **10%** |
| C1C5 | sub-fslAchieva04 | 0.772 | 0.69 | 0.861 | 0.555 | 0.861 | 0.74 | 0.74 | 0.756 | 0.772 |
|  | sub-mgh04 | 0.606 | 0.476 | 0.229 | 0.126 | 0.126 | 0.082 | 0.126 | 0.093 | 0.089 |
|  | sub-mni01 | 0.219 | 0.302 | 0.201 | 0.201 | 0.258 | 0.219 | 0.193 | 0.228 | 0.238 |
|  | sub-perform05 | 0.795 | 0.276 | 0.253 | 0.176 | 0.184 | 0.184 | 0.212 | 0.138 | 0.16 |
|  | sub-sherbrooke02 | 1.0 | 0.881 | 0.894 | 0.894 | 0.853 | 0.881 | 0.881 | 0.853 | 0.894 |
|  | sub-stanford02 | 1.0 | 0.44 | 0.352 | 0.232 | 0.275 | 0.212 | 0.275 | 0.232 | 0.202 |
| C1C2 | sub-fslAchieva04 | 0.449 | 0.185 | 0.275 | 0.082 | 0.185 | 0.143 | 0.099 | 0.109 | 0.091 |
|  | sub-mgh04 | 0.985 | 0.758 | 0.871 | 0.733 | 0.708 | 0.733 | 0.631 | 0.733 | 0.682 |
|  | sub-mni01 | 0.542 | 0.183 | 0.331 | 0.214 | 0.198 | 0.183 | 0.214 | 0.156 | 0.183 |
|  | sub-perform05 | 0.965 | 0.748 | 0.811 | 0.934 | 0.867 | 0.811 | 0.867 | 0.892 | 0.84 |
|  | sub-sherbrooke02 | 1.0 | 0.922 | 0.901 | 0.823 | 0.823 | 0.901 | 0.823 | 0.823 | 0.851 |
|  | sub-stanford02 | 0.823 | 0.851 | 0.598 | 0.73 | 0.698 | 0.665 | 0.73 | 0.698 | 0.665 |
| C2C5 | sub-fslAchieva04 | 0.882 | 0.378 | 0.233 | 0.265 | 0.195 | 0.392 | 0.265 | 0.254 | 0.276 |
|  | sub-mgh04 | 0.93 | 0.432 | 0.328 | 0.206 | 0.197 | 0.316 | 0.242 | 0.262 | 0.242 |
|  | sub-mni01 | 0.974 | 0.937 | 0.654 | 0.515 | 0.636 | 0.689 | 0.672 | 0.672 | 0.707 |
|  | sub-perform05 | 0.881 | 0.867 | 0.867 | 0.56 | 0.836 | 0.693 | 0.693 | 0.617 | 0.636 |
|  | sub-sherbrooke02 | 0.999 | 0.955 | 0.798 | 0.78 | 0.903 | 0.89 | 0.903 | 0.89 | 0.877 |
|  | sub-stanford02 | 0.999 | 0.742 | 0.502 | 0.611 | 0.649 | 0.63 | 0.63 | 0.593 | 0.434 |

**Supplementary Table 2.** Measurement error of the estimated atrophy using Jim’s AS on synthetic images in the PAM50 template. The numerical values in the table represent the difference between measured and simulated atrophy. The error of the measured atrophy from CSA estimates is presented for various levels of simulated atrophy under different noise conditions. Analyses were conducted for the cervical segments C1C2 and C2C5. A positive error denotes overestimation, while a negative error signifies underestimation of the expected atrophy level.

| **Cervical level** | **Noise level (%)** | **Measurement error of estimated atrophy by percent simulated atrophy** | | | | | | | | | |
| --- | --- | --- | --- | --- | --- | --- | --- | --- | --- | --- | --- |
|  |  | **0%** | **0.5%** | **1%** | **1.5%** | **2%** | **3%** | **4%** | **5%** | **6%** | **10%** |
| C1C2 | 0 | -0.01 | -0.18 | -0.31 | -0.43 | -0.54 | -0.77 | -0.99 | -1.2 | -1.38 | -1.79 |
|  | 1 | -0.02 | -0.2 | -0.34 | -0.39 | -0.56 | -0.76 | -1.01 | -1.19 | -1.37 | -1.76 |
|  | 2 | 0.08 | -0.22 | -0.34 | -0.53 | -0.53 | -0.81 | -0.89 | -1.19 | -1.3 | -1.86 |
|  | 4 | -0.1 | -0.36 | -0.27 | -0.15 | -0.7 | -0.91 | -1.05 | -1.16 | -1.35 | -1.8 |
|  | 8 | -0.35 | -0.37 | -0.33 | -0.68 | -0.47 | -1.03 | -1.22 | -1.64 | -1.56 | -2.0 |
| C2C5 | 0 | -0.25 | 0.02 | 0.06 | 0.12 | 0.2 | 0.31 | 0.42 | 0.63 | 0.79 | 1.31 |
|  | 1 | -0.29 | 0.01 | 0.01 | 0.13 | 0.13 | 0.34 | 0.41 | 0.64 | 0.82 | 1.27 |
|  | 2 | -0.29 | 0.04 | 0.02 | 0.03 | 0.24 | 0.32 | 0.52 | 0.55 | 0.7 | 1.37 |
|  | 4 | -0.2 | 0.12 | 0.22 | 0.15 | -0.04 | 0.34 | 0.78 | 0.33 | 0.54 | 1.11 |
|  | 8 | -0.65 | -0.13 | 0.13 | -0.37 | 0.21 | -0.28 | 0.52 | -0.4 | -0.17 | 0.64 |

**Supplementary Table 3.** Measurement error of the estimated atrophy using Jim’s Reg on synthetic images in the PAM50 template. The numerical values in the table represent the difference between measured and simulated atrophy. The error of the measured atrophy from CSA estimates is presented for various levels of simulated atrophy under different noise conditions. Analyses were conducted for the cervical segments C1C2 and C2C5. A positive error denotes overestimation, while a negative error signifies underestimation of the expected atrophy level.

| **Cervical level** | **Noise level (%)** | **Measurement error of estimated atrophy by percent simulated atrophy** | | | | | | | | | |
| --- | --- | --- | --- | --- | --- | --- | --- | --- | --- | --- | --- |
|  |  | **0%** | **0.5%** | **1%** | **1.5%** | **2%** | **3%** | **4%** | **5%** | **6%** | **10%** |
| C1C2 | 0 | 0.0 | -0.21 | -0.19 | -0.14 | -0.16 | -0.22 | -0.21 | -0.24 | -0.28 | -0.53 |
|  | 1 | 0.01 | -0.21 | -0.16 | -0.04 | -0.13 | -0.23 | -0.19 | -0.17 | -0.26 | -0.46 |
|  | 2 | 0.04 | -0.23 | -0.27 | -0.25 | -0.28 | -0.3 | -0.14 | -0.26 | -0.26 | -0.61 |
|  | 4 | -0.09 | -0.43 | -0.11 | -0.06 | -0.32 | -0.4 | -0.05 | -0.32 | -0.23 | -0.74 |
|  | 8 | -0.16 | 0.04 | 0.14 | -0.14 | 0.13 | -0.27 | -0.24 | 0.03 | 0.18 | -0.26 |
| C2C5 | 0 | -0.07 | -0.31 | -0.33 | -0.43 | -0.6 | -0.74 | -0.94 | -1.15 | -1.3 | -1.7 |
|  | 1 | -0.12 | -0.29 | -0.37 | -0.51 | -0.76 | -0.77 | -0.99 | -1.16 | -1.32 | -1.71 |
|  | 2 | -0.08 | -0.24 | -0.35 | -0.52 | -0.52 | -0.87 | -0.96 | -1.21 | -1.33 | -1.56 |
|  | 4 | -0.13 | -0.17 | -0.29 | -0.43 | -0.88 | -0.67 | -0.87 | -1.07 | -1.24 | -1.78 |
|  | 8 | -0.48 | -0.41 | -0.31 | -0.6 | -0.78 | -1.11 | -1.28 | -1.57 | -1.73 | -1.83 |

**Supplementary Table 4.** Root Mean Square Error (RMSE) of the estimated atrophy using Jim’s AS on synthetic images generated from real T1-w images of six randomly selected subjects from the *spine generic* dataset. The RMSE of the measured atrophy from CSA estimates is presented for various levels of simulated atrophy under different noise conditions. Analyses were conducted for the cervical segments C1C2 and C2C5 The RMSE calculation involved first computing the squared differences between the measured and reference values for each subject, averaging these squared differences, and then taking the square root of the average. This approach quantifies the overall deviation, with lower RMSE values indicating higher accuracy.

| **Cervical level** | **Noise level (%)** | **RMSE of estimated atrophy by percent simulated atrophy** | | | | | | | | | |
| --- | --- | --- | --- | --- | --- | --- | --- | --- | --- | --- | --- |
|  |  | **0%** | **0.5%** | **1%** | **1.5%** | **2%** | **3%** | **4%** | **5%** | **6%** | **10%** |
| C1C2 | 0 | 0.21 | 0.22 | 0.35 | 0.34 | 0.30 | 0.66 | 0.76 | 0.93 | 0.94 | 1.22 |
|  | 1 | 0.23 | 0.22 | 0.37 | 0.34 | 0.47 | 0.68 | 0.80 | 0.96 | 1.03 | 1.32 |
|  | 2 | 0.32 | 0.35 | 0.33 | 0.31 | 0.31 | 0.49 | 0.66 | 0.72 | 0.94 | 1.20 |
|  | 4 | 0.29 | 0.36 | 0.39 | 0.31 | 0.51 | 0.57 | 0.76 | 0.86 | 1.13 | 1.11 |
|  | 8 | 0.28 | 0.57 | 0.69 | 0.75 | 0.83 | 0.95 | 1.02 | 1.16 | 1.17 | 1.27 |
| C2C5 | 0 | 0.17 | 0.22 | 0.36 | 0.24 | 0.11 | 0.23 | 0.24 | 0.43 | 0.41 | 0.75 |
|  | 1 | 0.14 | 0.23 | 0.38 | 0.24 | 0.23 | 0.28 | 0.32 | 0.47 | 0.48 | 0.80 |
|  | 2 | 0.20 | 0.29 | 0.37 | 0.24 | 0.23 | 0.30 | 0.40 | 0.41 | 0.46 | 0.75 |
|  | 4 | 0.18 | 0.32 | 0.41 | 0.37 | 0.30 | 0.32 | 0.54 | 0.67 | 0.57 | 0.80 |
|  | 8 | 0.33 | 0.32 | 0.65 | 0.57 | 0.35 | 0.52 | 0.55 | 0.75 | 0.79 | 0.94 |

**Supplementary Table 5.** Root Mean Square Error (RMSE) of the estimated atrophy using Jim’s Reg on synthetic images generated from real T1-w images of six randomly selected subjects from the *spine generic* dataset. The RMSE of the measured atrophy from CSA estimates is presented for various levels of simulated atrophy under different noise conditions. Analyses were conducted for the cervical segments C1C2 and C2C5 The RMSE calculation involved first computing the squared differences between the measured and reference values for each subject, averaging these squared differences, and then taking the square root of the average. This approach quantifies the overall deviation, with lower RMSE values indicating higher accuracy.

| **Cervical level** | **Noise level (%)** | **RMSE of estimated atrophy by percent simulated atrophy** | | | | | | | | | |
| --- | --- | --- | --- | --- | --- | --- | --- | --- | --- | --- | --- |
|  |  | **0%** | **0.5%** | **1%** | **1.5%** | **2%** | **3%** | **4%** | **5%** | **6%** | **10%** |
| C1C2 | 0 | 0.02 | 0.26 | 0.31 | 0.41 | 0.57 | 0.56 | 0.85 | 0.76 | 1.09 | 1.76 |
|  | 1 | 0.05 | 0.26 | 0.32 | 0.45 | 0.50 | 0.56 | 0.70 | 0.77 | 0.81 | 1.01 |
|  | 2 | 0.08 | 0.23 | 0.34 | 0.37 | 0.44 | 0.53 | 0.59 | 0.74 | 0.79 | 1.00 |
|  | 4 | 0.03 | 0.22 | 0.30 | 0.47 | 0.57 | 0.86 | 0.87 | 0.98 | 1.10 | 1.26 |
|  | 8 | 0.25 | 0.52 | 0.38 | 0.53 | 0.56 | 0.71 | 0.73 | 0.90 | 0.77 | 0.85 |
| C2C5 | 0 | 0.03 | 0.20 | 0.33 | 0.40 | 0.29 | 0.64 | 0.39 | 0.85 | 0.52 | 1.12 |
|  | 1 | 0.06 | 0.19 | 0.31 | 0.39 | 0.44 | 0.64 | 0.73 | 0.87 | 0.90 | 1.50 |
|  | 2 | 0.11 | 0.11 | 0.24 | 0.32 | 0.42 | 0.59 | 0.70 | 0.80 | 0.90 | 1.53 |
|  | 4 | 0.15 | 0.22 | 0.23 | 0.34 | 0.44 | 0.50 | 0.70 | 0.91 | 0.91 | 1.62 |
|  | 8 | 0.38 | 0.35 | 0.40 | 0.54 | 0.56 | 0.86 | 0.90 | 1.21 | 1.23 | 1.73 |


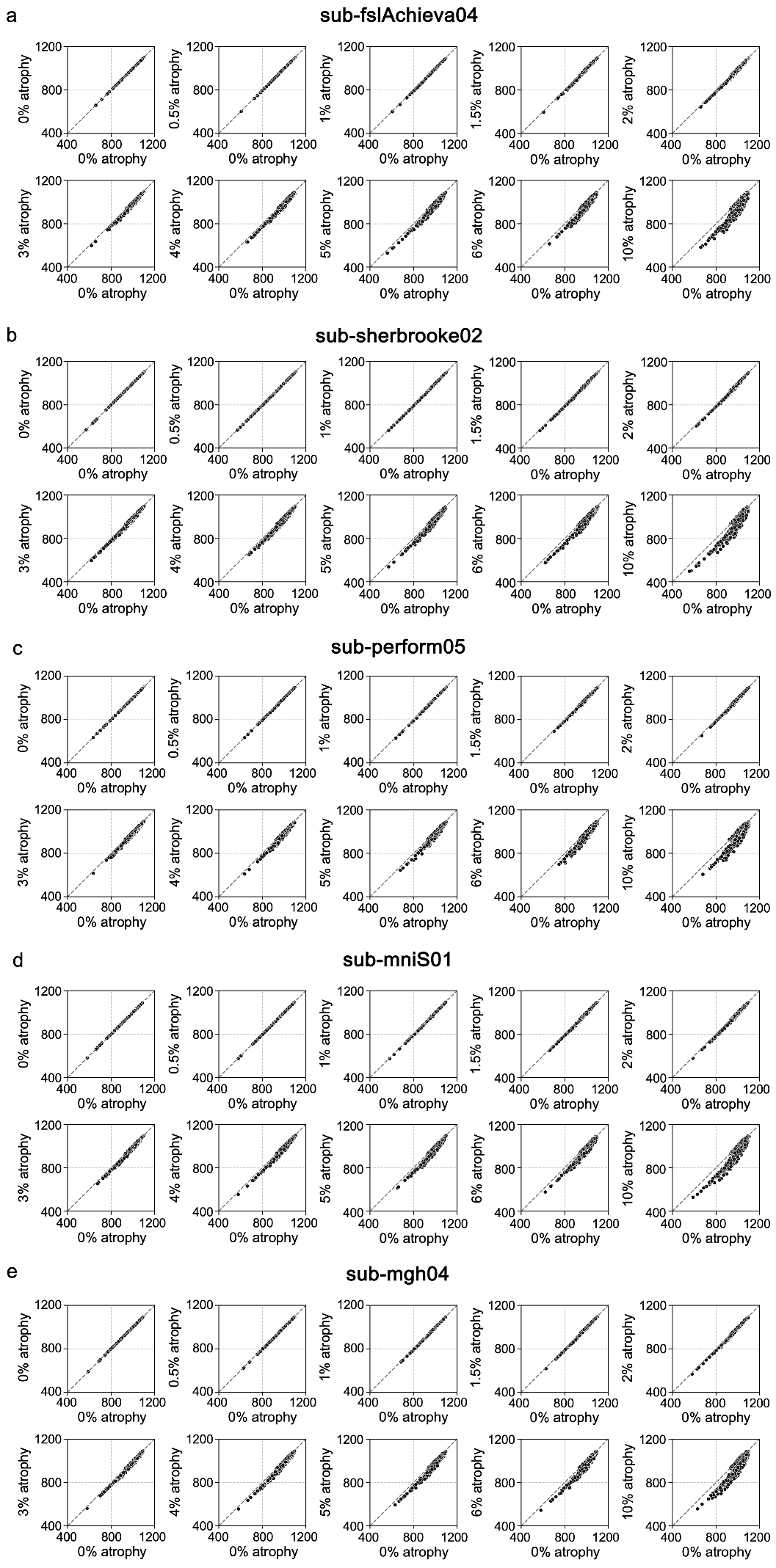


**Supplementary Figure 1.** Characterization of the intensity distribution for different levels of simulated atrophy in comparison to 0% atrophy for the remaining subjects extracted from the *spine generic* dataset.


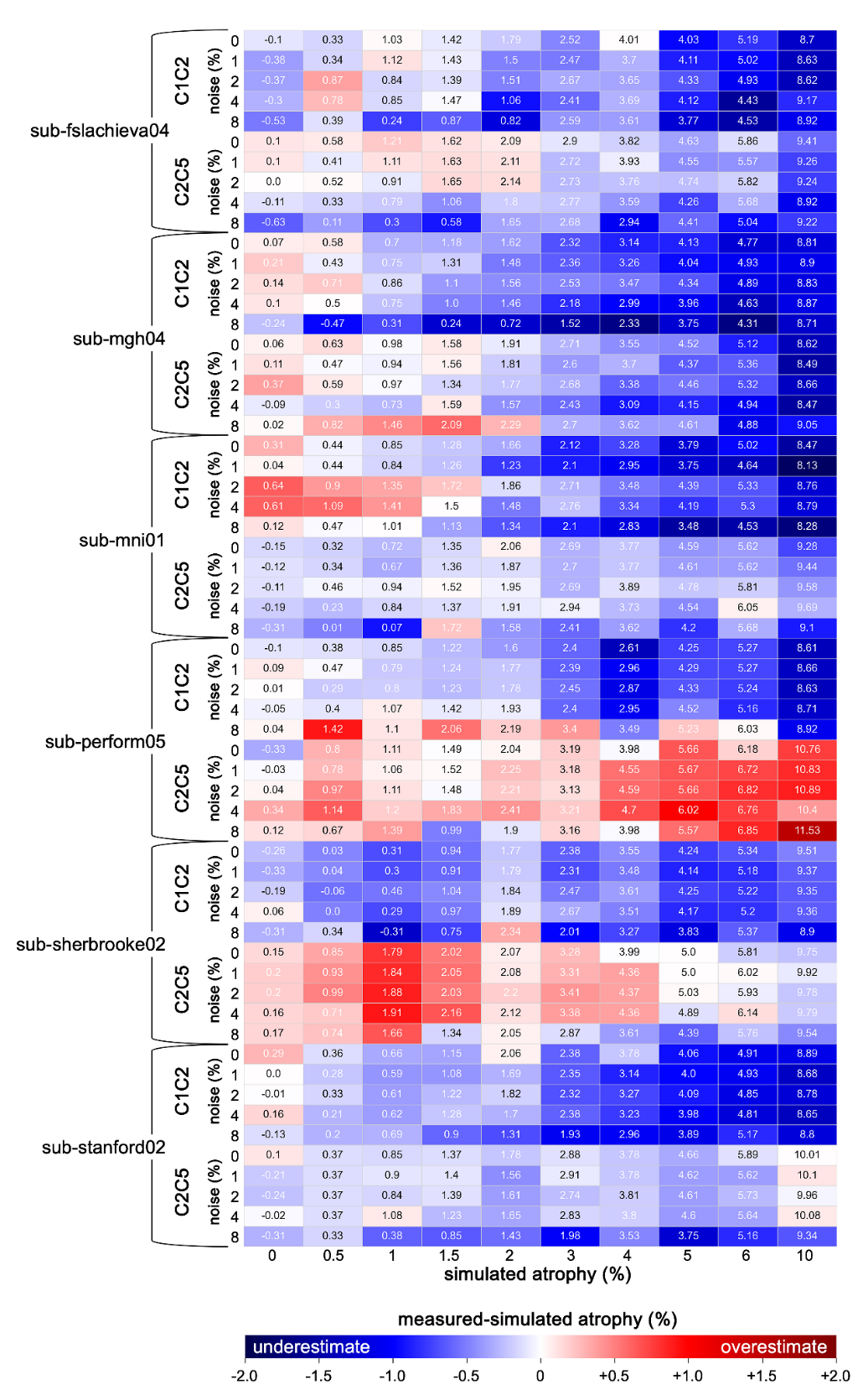


**Supplementary Figure 2.** Estimated atrophy using Jim’s AS on synthetic images generated from real T1-w images of six randomly selected subjects from the *spine generic* dataset, along with measurement error (color bar). The numerical values in the color matrix denote the measured atrophy levels and the color shades visually represent the magnitude of the error (measured−simulated atrophy). The measured atrophy from CSA estimates is presented for various levels of simulated atrophy under different noise conditions. Analyses were conducted for the cervical segments C1C2 and C2C5. A positive error denotes overestimation, while a negative error signifies underestimation of the expected atrophy level.


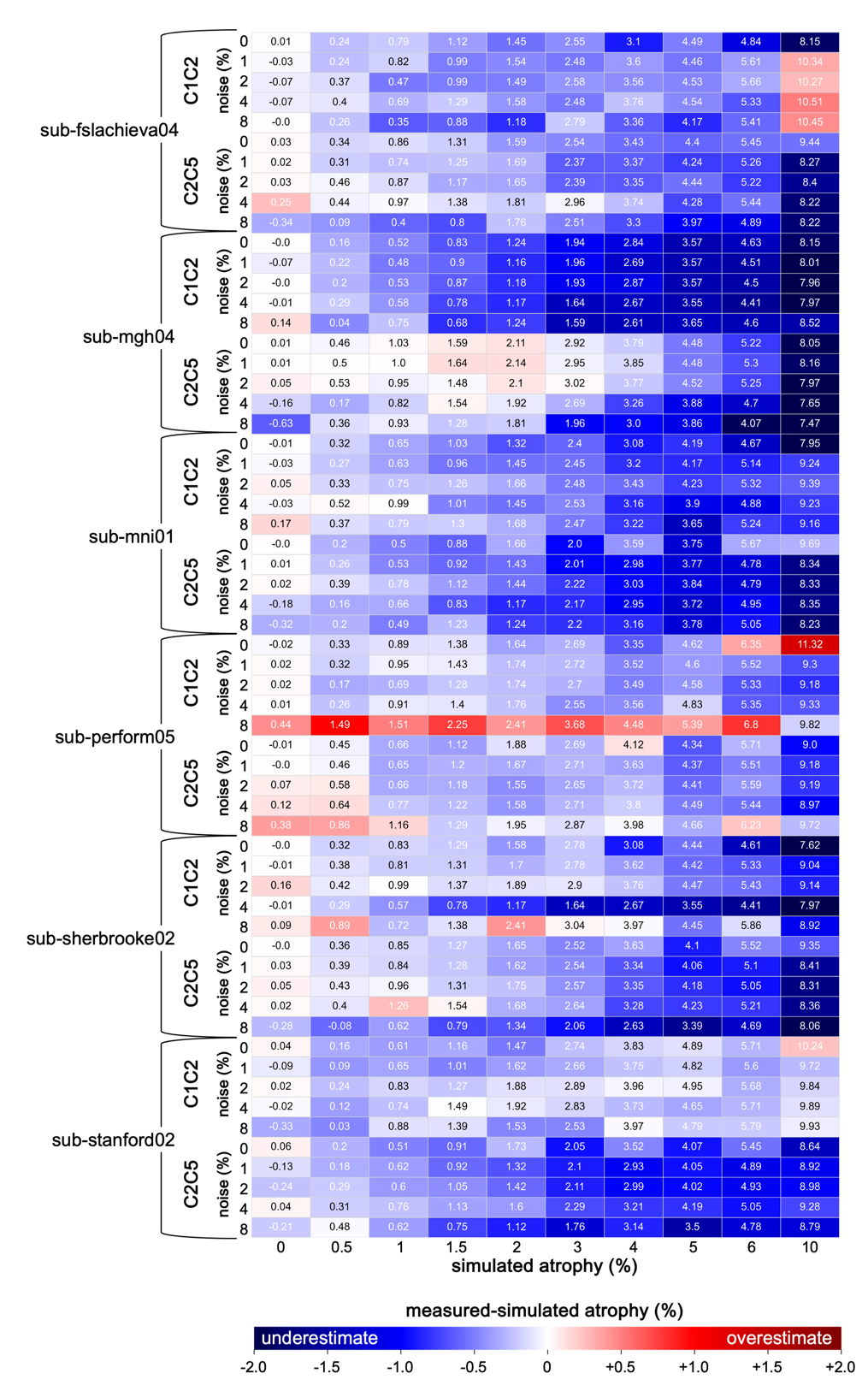


**Supplementary Figure 3.** Estimated atrophy using Reg on synthetic images generated from real T1-w images of six randomly selected subjects from the *spine generic* dataset, along with measurement error (color bar). The numerical values in the color matrix denote the measured atrophy levels and the color shades visually represent the magnitude of the error (measured−simulated atrophy). The measured atrophy from CSA estimates is presented for various levels of simulated atrophy under different noise conditions. Analyses were conducted for the cervical segments C1C2 and C2C5. A positive error denotes overestimation, while a negative error signifies underestimation of the expected atrophy level.
